# Diversity and compositional differences in the oral microbiome of oral squamous cell carcinoma patients and healthy controls: a scoping review

**DOI:** 10.3389/froh.2024.1366153

**Published:** 2024-06-11

**Authors:** M. C. van Dijk, J. F. Petersen, J. E. Raber-Durlacher, J. B. Epstein, A. M. G. A. Laheij

**Affiliations:** ^1^ Department of Oral Medicine, Academic Centre for Dentistry Amsterdam, University of Amsterdam and VU University, Amsterdam, Netherlands; ^2^ Department of Oral and Maxillofacial Surgery, Amsterdam UMC, University of Amsterdam, Amsterdam, Netherlands; ^3^ City of Hope Comprehensive Cancer Center, Duarte CA and Samuel Oschin Comprehensive Cancer Institute, Cedars-Sinai Medical System, Los Angeles, CA, United States

**Keywords:** oral cancer, oral microbiome, oral squamous cell carcinoma (OSCC), microbial diversity, taxonomic level, *Fusobacterium nucleatum*, *Streptococcus*

## Abstract

**Objectives:**

The human oral microbiome may play a role in the development of oral squamous cell carcinoma. The aim of this scoping review was to examine microbial diversity and differences in the composition of the oral microbiome between OSCC patients and healthy controls.

**Methods:**

A literature search (in PubMed and Embase.com) was performed on January 9, 2023. The outcome variables used from the included studies of this review were alpha- and beta diversity and oral microbiome composition profiles for each taxonomic level (phylum-, class-, order-, genus- and species level).

**Results:**

Thirteen out of 423 studies were included in this review compromising 1,677 subjects, of which 905 (54.0%) were OSCC patients and 772 (46.0%) were healthy controls. Most studies found a higher alpha diversity in the OSCC patient group and significantly different beta diversities between OSCC patient samples and healthy control samples. Studies reported more abundant *Fusobacteria* (on phylum level), *Fusobacterium* (on genus level), *Fusobacterium nucleatum, Porphyromonas endodontalis* and *Prevotella intermedia* (on species level) in OSCC patients. The healthy control group had more abundant *Actinobacteria* (on phylum level), *Streptococcus* and *Veilonella* (on genus level) and *Veilonella parvula* (on species level) according to most studies.

**Conclusions:**

Our findings show differences in oral microbiome diversity and composition in OSCC patients. Clinical implications demand continuing study. Development of internationally accepted standard procedures for oral sample collection and oral microbiota analysis is needed for more conclusive and clinically relevant comparisons in future research.

## Introduction

Oral squamous cell carcinoma (OSCC) is the most frequent malignancy in the oral cavity and one of the 10 most common cancers worldwide (1). Identified risk factors of OSCC include tobacco use, alcohol use and areca nut intake (2). Human papillomavirus (HPV) may also have oral malignant potential in OSCC development, specifically in younger patients without exposure to the main risk factors (3), although increasing cases in younger adults without the above potential risk factors remain to be defined.

The human oral microbiome is a complex comprised of more than 700 different species (4). The oral microbiome can be classified in different taxonomic levels: phylum-, class-, order-, family-, genus- and species level. The healthy oral cavity can be broadly categorized into six phyla: *Firmicutes, Actinobacteria, Proteobacteria, Fusobacteria, Bacteroidetes* and *Spirochaetes* constituting 96% of total oral bacteria (5). New technologies like next-generation sequencing and metagenomic shotgun sequencing have revealed the complexity of the human oral microbiome (4).

Oral diseases such as caries, oral mucositis, gingivitis and periodontitis are linked to dysbiotic shifts of the oral microbiome (6–8). The ecosystem of the mouth can become dysbiotic due to salivary changes, poor oral hygiene and lifestyle factors like diet, smoking, disease and stress (4). In addition, systemic medications use such as antibiotics, prednisone, cancer chemotherapy, and topicals such as steroids may lead to microbial shifts (9, 10–13). To combat oral diseases, an approach to treatment may be to re-establish symbiosis of the oral microbiome (8, 14).

Evidence that members of the human oral microbiome may be associated oral cancer is growing (15). In OSCC, carcinogenesis is hypothesized to begin with an alteration of the oral microbiome composition due to risk factors of oral cancer, like alcohol intake and tobacco use, leading to chronic inflammation. Bacterial products and metabolic by-products of the altered oral microbiome can induce permanent genetic alterations in epithelial cells. Further, genetically altered epithelial cells proliferate and apoptosis is inhibited leading to dysplasia and neoplastic cell proliferation. Finally, tumour cells infiltrate surrounding tissues and eventually metastasize (16). In this hypothesis OSCC might arise from genetic alterations which can be induced by micro-organisms present in the oral microbiome (17).

Individual microorganisms are thought to contribute to cancer risk through various pathways including promoting cell proliferation and invasion, promoting metastasis, influencing the tumour immune microenvironment, increasing chemoresistance, promote chronic inflammation (18). The role of the oral microbiome in OSCC has been increasingly recognized and studied in individual studies (16, 18). However, it remains unclear whether individual microorganisms or microbial signatures can be linked to OSCC. Therefore, the aim of this scoping review is to compare the microbial diversity and oral microbiome composition differences between OSCC patients and healthy controls.

## Methods

A literature search was performed based on the Preferred Reporting Items for systematic Reviews and Meta-Analyses (PRISMA) statement (19). To identify all relevant publications, a systematic search in the bibliographic databases PubMed and EMBASE was conducted from inception to January 9, 2023. In PubMed, the following strings were combined: “Oral microbiome”[tiab] OR “mouth microbiome”[tiab] OR “oral bacteria”[tiab] OR “mouth bacteria”[tiab] OR “Microbiota”[Mesh] AND “Oral cancer”[tiab] OR “mouth cancer”[tiab] OR “cancer of the mouth”[tiab] OR “oral tumor”[tiab] OR “oral tumour”[tiab] OR “mouth tumor”[tiab] OR “mouth tumour”[tiab] OR “Mouth Neoplasms”[Mesh]. In EMBASE, the combined strings were: “Oral microbiome”:ti,ab,kw OR “mouth microbiome”:ti,ab,kw OR “oral bacteria”:ti,ab,kw OR “mouth bacteria”:ti,ab,kw OR “oral microbiome”/exp OR “mouth flora”/exp AND “oral cancer”:ti,ab,kw OR “mouth cancer”:ti,ab,kw OR “cancer of the mouth”:ti,ab,kw OR “oral tumor”:ti,ab,kw OR “oral tumour”:ti,ab,kw OR “mouth tumor”:ti,ab,kw OR “mouth tumour”:ti,ab,kw OR “mouth tumor”/exp OR “mouth cancer”/exp. Specific searches entered in PubMed and EMBASE are added in the Supplementary Appendix File.

The potentially relevant titles and abstracts yielded from the search were screened for eligibility using review manager Rayyan (20). Studies were included that met the following criteria: (a) studies that compare the oral microbiome of OSCC diagnosed patients with healthy controls; (b) observational studies (case-control, cohort, and cross-sectional studies); (c) written in English or Dutch. Studies were excluded if they were: (a) published before 2003; (b) *in vitro* studies, animal studies, letters or comments on articles, study protocols, preliminary studies, pilot studies, case series (<10 patients) or case reports.

The diversity of the oral microbiome composition in individual studies was reported as alpha- and/or beta diversity. Alpha diversity describes the species diversity/richness within a sample. For alpha diversity, different indices were used, including the Shannon-, Simpson-, InvSimpson-, Chao 1- and Faith's PD index. Beta diversity describes diversity between samples, in this case between OSCC patient samples and healthy control samples. To define beta diversity, Bray-Curtis dissimilarity was calculated. Data on oral microbiome composition profiles was categorized per taxonomic level, presented in relative abundance (%). For each taxonomic level (phylum-, class-, order-, genus- and species level), relative bacterial abundance differences between cases and controls were statistically tested in the individual studies. Only statistically significant differences (*p* < 0.05) were considered in this review.

## Results

**Figure 1:**
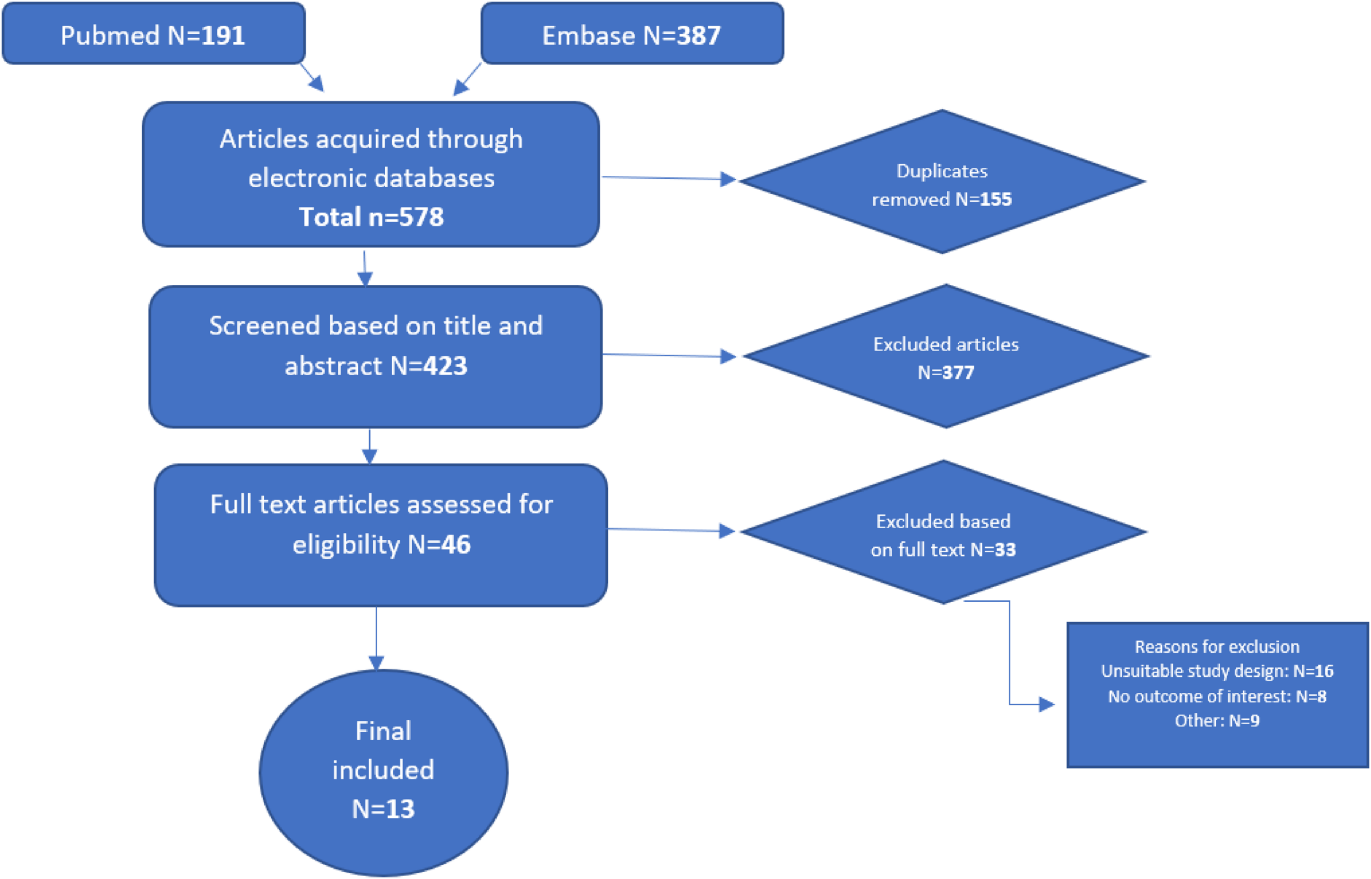
Flow diagram of the study selection process.

A total of 578 studies were retrieved, of which 155 duplicates were removed. After reading 423 abstracts, 46 full text studies were assessed for eligibility resulting in 13 studies included in this review (Figure 1).

A total of 1,677 subjects from 13 studies were included, of which 905 (54.0%) were OSCC patients and 772 (46.0%) were healthy controls. Of all subjects, 937 (55.9%) were male, 356 (21.2%) were female, and of 384 (22.9%) subjects the gender was unknown. From all subjects, 738 reported smoking (44.0%) and 571 (34.0%) reported regular alcohol consumption. The sampling methods were performed by saliva sampling or via oral swabs. The sequencing method for 9 included studies was performed via 16S rRNA sequencing. Of the remaining studies, two studies performed sequencing via 16S rDNA sequencing, the other two studies used metagenomic shotgun sequencing. An overview of the study characteristics is presented in Table 1.

**Table 1:** Characteristics of the studies included.

First author	Year	Study design	Population characteristics	*N* OSCC patients	*N* Healthy controls	Sampling method	Sequencing method	Outcomes	Other
Ganly	2022	Case-control	– Age: 63.0 ± 12.0 – Gender: 43M/44F – Smokers: 0 – Drinking: 21 – Ethnicity: 81.5% white, 18.5% other	42	45	Mouthwash sample collection	Metagenomic shotgun sequencing (MSS)	> Alpha and Beta diversity > Microbiome composition: phylum, genus and species level > Bacterial pathway analysis	Specifically in non-smoking volunteers
Liu	2022	Case-control	– Age: 61.0 ± 13.1 – Gender: 34M/16F – Smokers: 19 – Drinking: 11 – Ethnicity: unknown	40	10	Oral swab of the cancer site	Metagenomic analysis	> Beta diversity > Microbiome composition: phylum level, class level, species level	4 OSCC patient groups (*N* = 10) with different cancer invasion depths
Ueda	2021	Case-control	– Age ≥ 60 years: 72 – Gender: 62M/36F – Smokers: 14 – Drinking: 20 – Ethnicity: unknown	48	50	Saliva sample collection (spitting method)	Next generation sequencing (of bacterial 16S rRNA genes)	> Salivary microbiome composition: genus level > Salivary CCL20 levels	Main goal of the study was to investigate the salivary CCL20 level as a possible biomarker for OSCC
Zhou	2021	Case-control	– Age range: 34–78 years – Gender: unknown – No smoking or drinking – Ethnicity: unknown	47	48	Saliva sample collection and oral swabs of multiple sites in the oral cavity	16S rDNA sequencing	> Alpha and Beta diversity > Microbiome composition: phylum, class, order, family, genus and species level	Aim was to develop an early diagnostic model to screen OSCC based on the findings
Yang	2022	Case-control	– Age: 48.74 ± 13.23 – Gender: 11M/31F – Smokers: 7 – Drinking: 5 – Ethnicity: unknown	27	15	Oral swab of the cancer site	16S rRNA sequencing	> Alpha diversity > Microbiome composition: phylum and genus level	Correlation between variations in oral microbiota and tumour prediction
Hsiao	2018	Case-control	– Age: 54.6 ± 1.1 – Gender: unknown – Smokers: 163 – Drinking: 119 – Ethnicity: unknown	138	151	Saliva sample collection	16S rRNA sequencing	> Microbiome composition: species level and associated OSCC risk	Influences of lifestyle and genetic factors on OSCC risk are suggested by the found data
Granato	2021	Case-control	– Age: median 55 range (37–76) – Gender: 21M/3F – Smokers: 14 – Drinking: 14 – Ethnicity: unknown	16	8	Saliva sample collection	16S rDNA sequencing and meta-omics analysis	> Beta diversity > Microbiome composition: genus level and correlation with tumour characteristics and survival	The possible role of salivary proteins in OSCC is also researched
Kumar	2020	Cross-sectional prospective metagenomic study	– Age: 50.38 range (35–60) – Gender: 36M/13F – Smokers: 24 – Drinking: unknown – Ethnicity: unknown	25	24	Saliva sample collection	16S rRNA sequencing	> Alpha diversity > Microbiome composition: phylum, class, order, family, genus and species level	Salivary cytokine levels were also researched
Ganly	2019	Case-control	– Age: 52.10 ± 13.25 – Gender: 14M/16F – Smokers: 0 – Drinking: 11 – Ethnicity: unknown	18	12	Oral wash sample (rinse method)	16S rRNA gene sequencing	> Alpha and Beta diversity > Microbiome composition and analysis: genus level	Test group consists specifically of non-smoking, HPV-negative patients
Yang	2018	Cross-sectional	– Age: 47.93 ± 9.73 – Gender: 204M/44F – Smokers: 139 – Drinking: 110 – Ethnicity: unknown	197	51	Oral rinse sample	16S rRNA V3V4 amplicon sequencing	> Alpha and Beta diversity > Microbiome composition: phylum, genus and species level	Cancer progression stage (1 to 4) is also taken into consideration
Zhu	2022	Case-control	– Age > 60 years: 103 – Gender: 90M/88F – Smokers: 33 – Drinking: 32 – Ethnicity: unknown	70	108	Saliva sample collection	16S rRNA sequencing	> Alpha and Beta diversity > Microbiome composition: genus level, species level	Besides sequencing, the microbiome was also analysed by using fluorescence *in situ* hybridization, invasion and migration assays
Yang	2021	Cross-sectional	– Age: 52.56 ± 11.98 – Gender: 391M/37F – Smokers: 297 – Drinking: 196 – Ethnicity: unknown	208	220	Saliva sample collection	16S rRNA sequencing	> Alpha and Beta diversity > Microbiome composition: phylum and genus level	Host genetic associations with salivary microbiome were researched
Heng	2022	Case-control	– Age: 59.30 ± 10.61 – Gender: 31M/28F – Smokers: 28 – Drinking: 32 – Ethnicity: unknown	29	30	Saliva sample collection, buccal swabs and gingival plaque swabs	16S rRNA V3V4 amplicon sequencing	> Alpha diversity > Microbiome composition: phylum, genus and species level	Mycobiome was also researched

Alpha diversity from OSCC patients were compared to healthy controls in several studies. Alpha diversity was reported in nine of 13 included studies. In three studies, no significant difference in alpha diversity between groups was found (21–23). In five studies, alpha diversity was significantly higher in the OSCC patient group, compared to the healthy control group (24–28). On the other hand, in one study, the alpha diversity was significantly higher in the healthy control group, compared to the OSCC patient group (29).

Beta diversity was reported in eight studies. In six studies, beta diversity was significantly different between the OSCC patient group and controls (21, 23, 26–28, 30). One more study concluded a significant difference in beta diversity between groups, however, because of described population bias in this particular study, this result was omitted (24). In one study, no significant difference was found for the beta diversity between groups (26).

**Figure 2:**
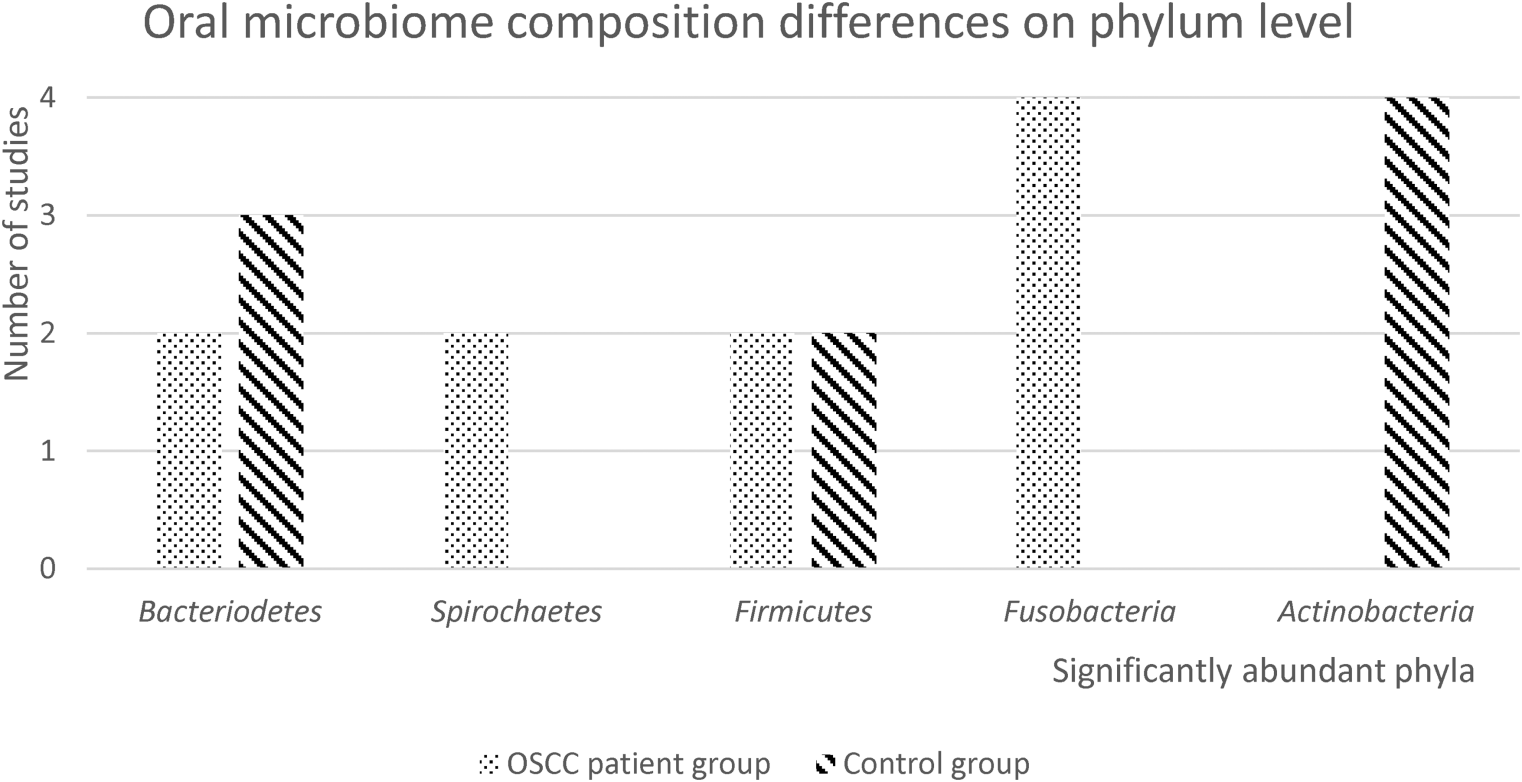
Oral microbiome composition differences on phylum level. In this figure, the number of studies with significantly abundant phyla in either the OSCC patient group or the healthy control group, is presented. Only significantly abundant phyla (*p* < 0.05), that were reported in 2 or more studies, are depicted.

Seven of the 13 included studies reported differences in the relative abundance of the oral microbiome between OSCC patient samples and control patients on phylum level (21, 24–28, 30) (Figure 2). Only phyla that were significantly different between patients and controls in two or more studies were included in this graph. Raw results for each taxonomic level can be found in the Supplementary Appendix.

Four studies found that *Fusobacteria* were significantly more abundant in OSCC patient samples, and four studies found that *Actinobacteria* were significantly more abundant in healthy control samples. *Firmicutes* were found to be significantly more abundant in both OSCC patient and control groups. Two studies reported that *Spirochaetes* were significantly more abundant in OSCC patient samples. Three studies found that *Bacteriodetes* were significantly more abundant in OSCC patient samples and two studies found they were more abundant in healthy control samples.

Differences between relative abundances of OSCC patient samples and healthy control samples on class level were reported in two out of the 13 included studies (24, 30). In one study, Flavobacteria and Spirochaetia were significantly more abundant in the OSCC patient group; Bacilli, Betaproteobacteria, Actinobacteria and Negativicutes were more abundant in the control group (30). In the other study, Negativicutes were significantly more abundant in the OSCC patient group; Bacilli, Bacterioidea and Betaproteobacteria were more abundant in the control group (24). On both family and order level, only one study reported data (24). Because of this limitation, results on family and order level were not included in this review.

Differences in the relative abundance at the genus level, were reported in 10 of the 13 included studies (21, 23–31).

**Figure 3:**
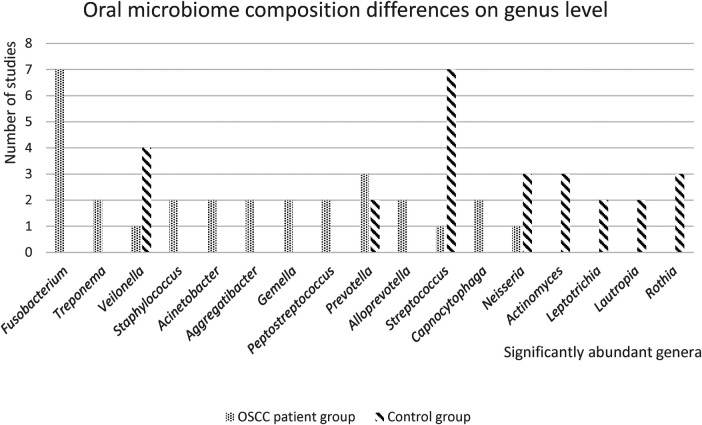
Oral microbiome differences on genus level. In this figure, the number of studies with significantly abundant genera in either the OSCC patient group or the healthy control group, is presented. Only significantly abundant genera (*p* < 0.05), that were reported in 2 or more studies, are depicted.

The oral microbiome composition differences on genus level are presented in Figure 3. The genus *Fusobacterium* was highly abundant in the OSCC patient group in most studies and the genus *Streptococcus* is highly abundant in the control group. Furthermore, genus *Veilonella* was mentioned in four studies as the significantly abundant genus in the healthy control group, while one study found that *Veilonella* was more abundant in the OSCC patient group. Eight out of the 13 included studies reported differences in relative abundance between OSCC patients and controls, on the species level (21, 22, 24, 26, 28–30, 32).

**Figure 4:**
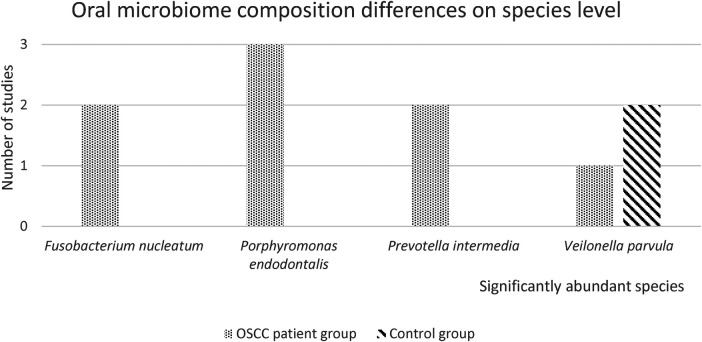
Oral microbiome differences on species level. In this figure, the number of studies with significantly abundant species in either the OSCC patient group or the healthy control group, is presented. Only significantly abundant species (*p* < 0.05), that were reported in 2 or more studies, are depicted.

The oral microbiome differences are presented in Figure 4. Only significantly abundant species that were mentioned in two or more studies were included. *Fusobacterium nucleatum* was significantly abundant in two studies, *Porphyromonas endodontalis* was significantly abundant in three studies and *Prevotella* intermedia was significantly abundant in two studies for the OSCC patient group. *Veilonella parvula* was significantly abundant in the OSCC patient group in one study, two studies reported *Veilonella parvula* was significantly more abundant in the control samples.

The results of the quality appraisal are presented in Table 2. A guideline for scoring each category was used and is described in more detail in the Supplementary Appendix. The majority of the studies had a case-control study design and almost all studies scored well on the critical appraisal. For one study, the risk of bias was reviewed as moderate (26). The main reason for the higher risk of bias, was the fact that in this study, the samples derived from the healthy controls were not matched to the OSCC patient group with respect to age, gender and oral health status.

**Table 2:** Quality assessment table of the studies included.

Study	Study eligibility	Study selection	Data collection	Synthesis and findings	Review risk of bias
Ganly et al. (21)	Low	Moderate	Low	Low	Low
Liu et al. (22)	Low	Moderate	Low	Low	Low
Ueda et al. (23)	Moderate	Low	Low	Low	Low
Zhou et al. (24)	Low	Low	Moderate	Low	Low
Yang et al. (25)	Low	Low	Low	Moderate	Low
Hsiao et al. (27)	Moderate	Low	Low	Low	Low
Granato et al. (26)	Low	Moderate	Low	Low	Low
Kumar et al. (28)	Moderate	Low	Low	Low	Low
Ganly et al. (29)	Low	Low	Low	Low	Low
C. Yang et al. (30)	Low	Moderate	Moderate	Low	Moderate
Zhu et al. (31)	Low	Low	Low	Low	Low
S. Yang et al. (32)	Moderate	Low	Low	Low	Low
Heng et al. (33)	Low	Low	Low	Low	Low

## Discussion

It has been hypothesized that OSCC might arise from genetic alterations induced by micro-organisms present in the oral microbiome (17). To gain more insight into which microbial signatures are linked to OSCC, the diversity and oral microbiome composition differences between OSCC patients and healthy controls were compared in this scoping review. A total of 13 studies were included in which oral microbial diversity (alpha- and beta diversity) and oral microbiome compositions (at different taxonomic levels) were compared between OSCC patients and healthy controls.

Most studies found a higher alpha diversity in the OSCC patient group and significantly different beta diversities in OSCC patient samples. Most studies concluded more abundant *Fusobacteria* (on phylum level), *Fusobacterium* (on genus level), *Fusobacterium nucleatum, Porphyromonas endodontalis* and *Prevotella intermedia* (on species level) in the OSCC patient group. The healthy control group had more abundant *Actinobacteria* (on phylum level), *Streptococcus* and *Veilonella* (on genus level) and *Veilonella parvula* (on species level) according to most studies.

Alpha diversity describes the species diversity/richness within a sample (33). Most studies found higher alpha diversity in the OSCC patient group. This was not expected because the general consensus is that less diversity in a microbiome indicates dysbiosis which is associated with pathological conditions such as caries, oral mucositis, periodontitis and even oral cancer (8, 34, 35). A possible explanation for this incoherent finding is the variety of sampling methods used in individual studies (35). In this review, some studies collected saliva samples, others took oral swabs of the cancer site or other sites in the oral cavity. This advocates for development of internationally accepted standard procedures for oral sample and metadata collection to promote more conclusive comparisons in future research (36). But even in homogeneous populations, high heterogeneity in microbiome diversity and composition is found (37). This complicates dividing populations into healthy and diseased based on their oral microbial diversity or composition differences.

Beta diversity describes diversity between samples, in this case between OSCC patient samples and healthy control samples (33). Beta diversity was significantly different between cases and controls in most studies, which may indicate a change in the microbiome composition in OSCC patients. This supports the potential contribution of the oral microbiome to OSCC development (16). Yet again, it is important to be cautious when using oral microbiome data to pinpoint differences between groups, because the oral microbiome is highly complex and the diversity measures may over-simplify this complexity (36). Also, different theories about the possible connection between microbiome changes and development of OSCC exist. Either bacteria may be the direct causative factor in the pathogenesis of OSCC and restructure the microbiome to an environment that damages healthy epithelial cells (bacteria before tumor theory), or bacterial presence in the OSCC tumor environment is opportunistic and is established after tumor development (bacteria after tumor theory) (38).

Most studies found *Fusobacteria* (on phylum level), *Fusobacterium* (on genus level), *Fusobacterium nucleatum, Porphyromonas endodontalis* and *Prevotella intermedia* (on species level) to be more abundant in the OSCC patient group. *F. nucleatum* is a Gram-negative, anaerobic oral bacterium that is a common inhabitant of the oral microbiome (39). In general, 5.2% of the healthy oral cavity constitutes of *Fusobacteria* (5). *F. nucleatum* is one of the main bacteria related to periodontitis and is recently associated to colorectal cancer and breast cancer (40, 41).

A comprehensive review of recent studies by McIlvanna showed *F. nucleatum* promotes several cancer development related mechanisms including activation of cell proliferation, promotion of cellular invasion, induction of chronic inflammation, and immune evasion (42). Li et al., described that *F. nucleatum* can promote oral squamous epithelial proliferation, metastasis, and immunomodulation via a large array of pathways (18). For instance, cells infected with *F. nucleatum* show a rise in gH2AX, a DNA double-strand break marker and a reduced expression of Ku70 and p53, both associated with cell repair (43). *F. nucleatum* promotes metastasis by activating EMT and the expression of MMPs (44). Moreover, it increases inflammation by influencing the AIM2 and POP1 pathway leading to the increased expression of IL-1B (18). Saikia et al., described the role of specific members of the oral microbiome, such as by influencing the defence mechanisms of oral mucosal stem cells and cancer stem cells (45). So, *F. nucleatum* has various ways of contributing to OSCC development.

In most studies, more abundant *Actinobacteria* (on phylum level), *Streptococcus* and *Veilonella* (on genus level) and *Veilonella parvula* (on species level) were found in the healthy control group compared to the OSCC patient group. *Actinobacteria* are ubiquitous gram-positive bacteria with high guanine and cytosine contents in DNA. *Actinobacteria* have a characteristic filamentous morphology (42). In general, 11.6% of the healthy oral cavity constitutes of *Actinobacteria.* Among *Firmicutes* (36.7% of oral bacteria), *Streptococcus* (19.2%) is the most abundant genus followed by *Veillonella* (8.6%) (5). *Streptococcus* species have been shown to impair *F. nucleatum*-induced inflammation in oral epithelial cells. A loss of *Streptococcus* species could therefore promote inflammation that is associated with *F. nucleatum* (42). What this example shows, is that loss of health associated species, can lead to growth of disease-associated species resulting in inflammation. After this dysbiotic shift in the microbiome and promoted inflammation, OSCC might arise via the hypothesized oral carcinogenesis pathway (16).

Besides studying microbiome makers for OSCC, previous studies used a wide range of salivary markers to detect OSCC including salivary lactate dehydrogenase (46), SLPI (47), salivary microRNA (48), salivary MMP-9 (49) and salivary sialic acid (50). Most studies included relatively small groups of OSCC patients, premalignant patients and controls. And none compared different promising biomarkers to determine relative predictive value of every biomarker. Other directions in research show promising results in predicting tumour progression including the intra-tumour microbiome, premalignant pathological characteristics, and premalignant genetic markers (51–53). In the future, large studies including already described salivary biomarkers, oral microbiome biomarkers and other biomarkers should be conducted to determine the most appropriate (combination) of biomarkers to predict OSCC. This could lead to the development of prediction models based on all risk factors, which might accurately estimate the risk of cancer development. Such prediction models should be AI aided, as the analysis of several biological markers and the determination of the relative risk is very complex.

Early diagnosis of OSCC plays a critical role in the treatment and prognosis of OSCC as early detection leads to less invasive treatment plans, better survival rates and better quality of life for patients (17, 54). Analyzing salivary samples to assess microbiome and salivary markers for the early diagnosis of OSCC is a potential option due to its ease of collection, non-invasiveness, and cost-efficiency when compared to traditional methods (24). The analysis of the oral microbiome in saliva samples may serve as a potential screening tool for detecting OSCC in the future, particularly in high-risk patients or those with premalignant lesions in the oral cavity. To accomplish this, larger longitudinal studies are required to track the oral microbiome composition of these patients over time, identifying those who develop OSCC and those who do not.

Specific outcomes of different studies are difficult to compare as there are many (small) differences in sampling and analysis leading to (slightly) different outcomes. This includes, but is not limited to, differences in the specific niches on the oral cavity, differences in methods of collection (rinse vs. swabs), differences in rRNA analysis protocols, different sequencing methods, differences in primers, differences in pipelines and differences in statistical packages. For future microbiome studies, it is important that differences in at least sampling methods are avoided and that standards are set.

To conclude, there are data on differences in the human oral microbiome between OSCC patients and healthy individuals. Higher microbial diversity was found in the healthy controls. Some species, e.g., *Streptococcus spp*, were associated with oral health, while *F. nucleatum* was associated with OSCC. Translating this data into clinically meaningful insights remains challenging. Further research is necessary to discover practical applications for this acquired knowledge in clinical settings (45, 55).
