# Supplementary material for: Diversity and compositional differences in the oral microbiome of oral squamous cell carcinoma patients and healthy controls: a scoping review
Source: Front Oral Health. 2024 Jun 11;5:1366153. doi: 10.3389/froh.2024.1366153 (PMC11196763; doi:10.3389/froh.2024.1366153)
Supplement: Supplementary file 2 [file Table2.docx]

*Table 2 Quality assessment table of the studies included*

| Study | Study Eligibility | Study selection | Data collection | Synthesis and findings | Review risk of bias |
| --- | --- | --- | --- | --- | --- |
| Ganly et al., 2022 | Low | Moderate | Low | Low | Low |
| Liu et al., 2022 | Low | Moderate | Low | Low | Low |
| Ueda et al., 2021 | Moderate | Low | Low | Low | Low |
| Zhou et al., 2021 | Low | Low | Moderate | Low | Low |
| Yang et al., 2022 | Low | Low | Low | Moderate | Low |
| Hsiao et al., 2018 | Moderate | Low | Low | Low | Low |
| Granato et al., 2021 | Low | Moderate | Low | Low | Low |
| Kumar et al., 2021 | Moderate | Low | Low | Low | Low |
| Ganly et al., 2019 | Low | Low | Low | Low | Low |
| C. Yang et al., 2018 | Low | Moderate | Moderate | Low | Moderate |
| Zhu et al., 2022 | Low | Low | Low | Low | Low |
| S. Yang et al., 2021 | Moderate | Low | Low | Low | Low |
| Heng et al., 2022 | Low | Low | Low | Low | Low |
