# Supplementary material for: Diversity and compositional differences in the oral microbiome of oral squamous cell carcinoma patients and healthy controls: a scoping review
Source: Front Oral Health. 2024 Jun 11;5:1366153. doi: 10.3389/froh.2024.1366153 (PMC11196763; doi:10.3389/froh.2024.1366153)
Supplement: Supplementary file 3 [file Datasheet1.docx]

# **Appendix**

| Number | Study |
| --- | --- |
| 1 | Ganly et al., 2022 |
| 2 | Liu et al., 2022 |
| 3 | Ueda et al., 2021 |
| 4 | Zhou et al., 2021 |
| 5 | J. Yang et al., 2022 |
| 6 | Hsiao et al., 2018 |
| 7 | Granato et al., 2021 |
| 8 | Kumar et al., 2021 |
| 9 | Ganly et al., 2019 |
| 10 | C. Yang et al., 2018a |
| 11 | Zhu et al., 2022 |
| 12 | S. Yang et al., 2021 |
| 13 | Heng et al., 2022 |

Appendix table 1 Numbering of the studies as referred to in the following microbial composition appendix tables 2,3,4 and 5.

## Raw results microbial composition phylum level

| Study | OSCC patient group | Healthy control group |
| --- | --- | --- |
| 1 | *Bacteriodetes* | *Actinobacteria* |
|  | *Synergistetes* | *Firmicutes* |
|  | *Spirochaetes* |  |
| 2 | *Spirochaetes* |  |
| 4 | *Firmicutes* | *Bacteriodetes* |
|  | *Fusobacteria* | *Probacteria* |
| 5 | *Proteobacteria* | *Bacteriodetes* |
| 10 | *Fusobacteria* | *Bacteriodetes* |
|  |  | *Actinobacteria* |
| 12 | *Firmicutes* | *Actinobacteria* |
|  | *Fusobacteria* | *Patesibacteria* |
| 13 | *Bacteriodetes* | *Firmicutes* |
|  | *Fusobacteria* | *Actinobacteria* |

Appendix table 2 Significantly more abundant phyla for the OSCC patient group and healthy control group as found within the studies

## Raw results microbial composition class, order and family level

| CLASS LEVEL | OSCC patient group | Healthy control group |
| --- | --- | --- |
| Study 2 | *Flavobacteria* | *Bacilli* |
|  | *Spirochaetia* | *Betaproteobacteria* |
|  |  | *Actinobacteria* |
|  |  | *Negativicutes* |
| Study 4 | *Negativicutes* | *Bacilli* |
|  |  | *Bacterioidea* |
|  |  | *Betaproteobacteria* |
| ORDER LEVEL | **OSCC patient group** | **Healthy control group** |
| Study 4 | *Selemonodales* | *Bacteriodales* |
|  |  | *Lactobacillales* |
|  |  | *Neisseriales* |
| FAMILY LEVEL | **OSCC patient group** | **Healthy control group** |
| Study 4 | *Veilonellaceae* | *Streptococcaceae* |
|  |  | *Prevotellaceae* |
|  |  | *Neisseriaceae* |

Appendix table 3 Significantly more abundant results on class, order and family level for the OSCC patient group and healthy control group as found within the studies

## Raw results microbial composition genus level

| Study | OSCC patient group | Healthy control group |
| --- | --- | --- |
| 1 | *Fusobacterium* | *Corynebacterium* |
|  |  | *Streptococcus* |
|  |  | *Actinomyces* |
|  |  | *Cryptobacterium* |
|  |  | *Selenomonas* |
| 3 | *Fusobacterium* | *Streptococcus* |
|  | *Porphyromonas* |  |
|  | *Treponema* |  |
| 4 | *Fusobacterium* | *Streptococcus* |
|  | *Veilonella* | *Neisseria* |
|  |  | *Prevotella* |
| 5 | *Treponema* | *Neisseria* |
|  | *Micrococcys* | *Veilonella* |
|  | *Pseudomonas* | *Streptococcus* |
|  | *Janthinobacterium* | *Leptotrichia* |
|  | *Parvimos* | *Lautropia* |
|  | *Loktanella* | *Sphingopyxis* |
|  | *Staphylococcus* | *Sphingobium* |
|  | *Acinetobacter* | *Tannerella* |
|  | *Catonella* | *Actinomyces* |
|  | *Aggregatibacter* | *Rothia* |
|  | *Propionibacterium* |  |
| 7 | *Abiotrophia* | *Veilonella* |
|  | *Acinetobacter* | *Rothia* |
|  | *Alloscordovia* | *Moryella* |
|  | *Dialister* | *Kingella* |
|  | *Gemella* | *Centipeda* |
|  | *Granulicatella* |  |
|  | *Peptostreptococcus* |  |
|  | *Selenomonas* |  |
|  | *Staphylococcus* |  |
|  | *Stenotrophomonas* |  |
| 9 | *Fusobacterium* | *Streptococcus* |
|  | *Prevotella* |  |
|  | *Alloprevotella* |  |
| 10 | *Fusobacterium* | *Streptococcus* |
|  |  | *Haemophilus* |
|  |  | *Porphyromonas* |
|  |  | *Actinomyces* |
| 11 | *Streptococcus* | *Neisseria* |
|  | *Capnocytophaga* | *Leptotrichia* |
|  | *Peptostreptococcus* | *Lautropia* |
|  | *Prevotella* | *Campylobacter* |
|  | *Actinobacillus* | *Rothia* |
|  | *Lactobacillus* |  |
| 12 | *Gemella* | *Veilonella* |
|  | *Fusobacterium* | *Prevotella* |
| 13 | *Alloprevotella* | *Streptococcus* |
|  | *Neisseria* | *Veilonella* |
|  | *Fusobacterium* |  |
|  | *Prevotella* |  |
|  | *Aggregatibacter* |  |
|  | *Capnocytophaga* |  |

Appendix table 4 Significantly more abundant genera for the OSCC patient group and healthy control group as found within the studies

## Raw results microbial composition species level

| Study | OSCC patient group | Healthy control group |
| --- | --- | --- |
| 1 | *Porphyromonas* | *Prevotella* |
|  |  | *Campilobacter gracilis* |
|  |  | *Leptotrichia Wadei* |
|  |  | *Veilonella Parvula* |
| 2 | *Fusobacterium nucleatum* |  |
|  | *capnocytophaga sputigena* |  |
|  | *porphyromonas endodontalis* |  |
|  | *gemella haemolysans* |  |
| 4 | *Veilonella* | *Haemophilus* |
| 6 | *Prevotella tannerae* | *Streptococcus tigurinus* |
|  | *Fusobacterium nucleatum* |  |
|  | *Prevotella intermedia* |  |
| 8 | *P. melaninogenica* | *Neissara subflava* |
|  | *Streptococcus agninosus* | *Veilonella dispar* |
|  | *Veilonella parvula* | *Rothia dentocariosa* |
|  | *Prevotella pallens* | *R. mucilaginosa* |
|  | *Porphyromonas endodontalis* |  |
|  | *Prevotella nanceiensis* |  |
|  | *Dialister sp* |  |
|  | *Campylobacter ureolyticus* |  |
|  | *Fusobacterium sp* |  |
|  | *P. nigrescens* |  |
|  | *Neisseria bacilliformis* |  |
|  | *Peptostreptococcus anaerobius* |  |
| 10 | *Fusobacterium periodonticum* | *S. mitis* |
|  | *Parvimonas micra* | *Haemophilus parainfluenzae* |
|  | *Streptococcus constellatus* | *Porphyromonas pasteri* |
|  | *Heamophilus influenza* | *Veilonella parvula* |
|  | *Filifactor alocis* | *Actinomyces odontolyticus* |
| 11 | *Capnocytophaga gingivalis* |  |
| 13 | *Prevotella intermedia* | *Streptococcus salivarius* |
|  | *Porphyromonas endodontalis* |  |

Appendix table 5 Significantly more abundant species for the OSCC patient group and healthy control group as found within the studies
